# SRμCT Reveals 3D Microstructural Alterations of the Vascular and Neuronal Network in a Rat Model of Chronic Compressive Thoracic Spinal Cord Injury

**DOI:** 10.14336/AD.2019.0529

**Published:** 2019-05-29

**Authors:** Liyuan Jiang, Yong Cao, Zhen Liu, Shuangfei Ni, Jun Liu, Yoon Ha, Zixiang Luo, Chengjun Li, Shaohua Liu, Jingsong Li, Xianzhen Yin, Tianding Wu, Hongbin Lu, Jianzhong Hu

**Affiliations:** ^1^Department of Spine Surgery, Xiangya Hospital, Central South University, Changsha, China.; ^2^Key Laboratory of Organ Injury, Aging and Regenerative Medicine of Hunan Province, Changsha, China.; ^3^The First Chenzhou People's Hospital, Chenzhou, China.; ^4^Department of Neurosurgery, Spine and Spinal Cord Institute, Yonsei University College of Medicine, Seoul, Korea.; ^5^Department of Spine Surgery, The Third Xiangya Hospital, Central South University, Changsha, China.; ^6^Center for Drug Delivery System, Shanghai Institute of Materia Medica, Chinese Academy of Sciences, Shanghai, China.; ^7^Department of Sports Medicine, Research Centre of Sports Medicine, Xiangya Hospital, Central South University, Changsha, China

**Keywords:** chronic spinal cord injury, neurovascular unit, spinal cord microvasculature, SRμCT, 3D

## Abstract

The complex pathology of chronic thoracic spinal cord compression involves vascular and neuroarchitectural repair processes that are still largely unknown. In this study, we used synchrotron radiation microtomography (SRμCT) to quantitatively characterize the 3D temporal-spatial changes in the vascular and neuronal network after chronic thoracic spinal cord compression in order to obtain further insights into the pathogenesis of this disease and to elucidate its underlying mechanisms. Direct 3D characterization of the spinal cord microvasculature and neural microstructure of the thoracic spinal cord was successfully reconstructed. The significant reduction in vasculature and degeneration of neurons in the thoracic spinal cord visualized via SRμCT after chronic compression were consistent with the changes detected by immunofluorescence staining. The 3D morphological measurements revealed significant reductions of neurovascular parameters in the thoracic spinal cord after 1 month of compression and became even worse after 6 months without relief of compression. In addition, the distinct 3D morphological twist and the decrease in branches of the central sulcal artery after chronic compression vividly displayed that these could be the potential triggers leading to blood flow reduction and neural deficits of the thoracic spinal cord. Our findings propose a novel methodology for the 3D analysis of neurovascular repair in chronic spinal cord compression, both qualitatively and quantitatively. The results indicated that compression simultaneously caused vascular dysfunction and neuronal network impairment, which should be acknowledged as concurrent events after chronic thoracic spinal cord injury. Combining neuroprotection with vasoprotection may provide promising therapeutic targets for chronic thoracic spinal cord compression.

Chronic progressive spinal cord compression, which results from progressive stenosis of the spinal canal, is very common in clinical settings, accounting for 30%-80% of cases of nontraumatic spinal cord injury [1-3]. It results from mechanical compression of the spinal cord that impairs motor and sensory functions insidiously and progressively. Diseases such as ossification of the posterior longitudinal ligament, ossification of yellow ligaments, and disc herniation can lead to chronic spinal cord compression [4, 5]. Age-related degeneration contributes to narrowing of the spinal canal, a common finding in spine imaging of the elderly, causing neurological dysfunction [6]. The precise pathological mechanisms of chronic spinal cord compression remain unclear, but progressive vascular dysfunction leading to local ischemia has been proposed as a possible contributor to neural deficits [7].

Microvasculature and neurons are anatomically closely tied and respond simultaneously in focal regions of ischemic injury. The neurovascular unit (NVU), which refers to intertwined connections between the vascular and neuronal networks, is considered to be the functional and structural unit that maintains normal homeostasis of neurological function in the central nervous system [8-10]. Previous studies have revealed that chronic spinal cord injury induces dramatic microenvironmental changes in the neural parenchyma, preventing spinal cord regeneration[11, 12]. Despite the growing understanding of neurogenesis, which is a potential approach for the treatment of ischemic injuries, the maintenance and protection of neuronal function to avoid attack is still challenging. Control and modulation of the regional blood supply seem promising as a treatment method for ischemic injuries, but clinical trials have not shown any benefit for traumatic spinal cord injuries [13]. Recent studies have provided evidence that disruption of neurons and vasculature should be acknowledged as concurrent events in neurological disorders [14]. This approach suggests that the responses of the vascular and neuronal networks could be coordinated, indicating the crucial importance of analyzing these responses simultaneously.

Since very few investigations have focused on chronic thoracic spinal cord compression injuries, far less is known about their course, treatment response, and recovery potential. Additionally, the 3D microstructural morphological changes in the vascular and neuronal networks during thoracic spinal cord compression have not been fully elucidated. The angioarchitecture and neuronal network of the mammalian spinal cord has unique 3D characteristics, and it is a highly organized structure within the central nervous system [15-17]. Therefore, 3D investigations of alterations in the neuronal and vascular networks would undoubtedly be helpful for gaining further insight into pathological processes and for developing effective strategies for the treatment of chronic thoracic spinal cord compression. Currently, most of the knowledge about the influence of compression on neurovascular changes comes from histological studies on spinal cord sections [18]. The microstructural features of neuro-vascular changes after chronic cervical cord compression have been partially revealed by electron microscopy [19]. The 3D morphology of the cervical spinal neurovascular network has not been explored. In addition, no evidence exists regarding 3D alterations of neurovascular morphology in response to thoracic spinal cord compression. Synchrotron radiation microto-mography (SRμCT) has been recognized as a powerful tool to explore the 3D structure of biospecimens across a large spatial range, down to submicron resolutions [20-26]. In a previous study, we reported the feasibility of using SRμCT to visualize and quantitatively analyze the 3D morphology of spinal cord microvasculature after acute injuries [21, 24, 27]. In the present study, SRμCT was used to characterize and quantify the microstructural features of the vascular and neuronal network changes in response to chronic thoracic spinal cord compression.

## MATERIALS AND METHODS

### Experimental animals and ethics statement

All animal protocols were approved by the Animal Ethics Committee of Central South University (Approval No. 20170213). Animal care and use were conducted in accordance with the guidelines of the Administration Committee of Affairs Concerning Experimental Animals in Hunan Province, China. A total of 72 adult male Sprague-Dawley rats (250-300?g) obtained from the Animal Center of Central South University were randomly divided into a control group (n=24) with immune-fluorescence staining (n=8), SRμCT study for vasculature (n=8) and neuronal network(n=8); a 1 month postcompression surgery group (n=24); and a 6 months postcompression surgery group (n=24).

### Establishment of a rat model of chronic thoracic spinal cord compression 

Rats were anesthetized with a single intra-peritoneal injection of xylazine hydrochloride (4.5 mg/kg) and ketamine hydrochloride solution (90 mg/kg) (Sigma, K-113, USA), followed by skin preparation. With the rats in the prone position, an incision was made along the spine at level T10. Then, the interspinal ligament, supraspinal ligament, and paraspinous muscle tissue from T9 to T11 were stripped, the T10 spinous process and T10 partial lamina were removed to access the epidural space, and a water-absorbable polyurethane polymer (Fischer Chemical Co., USA) with the proper size (1×1×1 mm) was implanted into the T10 epidural space on the posterior median side of the spine to induce compression after expansion of the material, as previously described [28]. In the sham control group, the T10 laminae were removed without implantation of the material to induce compression. After surgery, the incision was closed, with the muscles and skin tightly sutured. All animals received a subcutaneous injection of penicillin G (8000 U/100 g) for 5 days postsurgery to prevent infection. For postoperative analgesia, a subcutaneous injection of buprenorphine (0.01 mg/kg) was administered twice a day for 5 days. After surgery, all animals were individually housed in cages in a temperature-controlled room with a 12-hour light/dark cycle and allowed free access to food and water.

### Immunostaining and neurovascular counting

Rats placed under deep anesthesia with a ketamine/ xylazine hydrochloride solution (90?mg and 4.5?mg/kg, respectively; intraperitoneal injection) were transcardially perfused with physiological saline containing heparin (50 IU/mL), followed by 200 mL of formalin-picric solution for fixation (4% formaldehyde, pH 7.4) at 1 and 6 months postsurgery. Thoracic spinal cord segments (T8-T10) were carefully harvested and fixed with 4% paraformaldehyde for another 12 hours. The next day the spinal cord segments were moved to 30% (w/v) sucrose (Merck Millipore) for cryoprotection and left at 4°C for 48 hours, moulded in Tissue-Tek ® (Sakura) and stored at -20°C. Longitudinal spinal cord sections (10 µm) were cut at -20°C using a Leica CM1900 cryostat (Leica Biosystems) and the sections were stored at -20°C until use. Hereafter, the spinal cord sections were washed in Tris-buffered saline (TBS; pH 7.4) and incubated with a blocking buffer in a solution of TBS containing 0.3% Triton X-100 (Applichem) and 1% bovine serum albumin (BSA; Sigma). Following a ten minutes washing, the sectionswere incubated with primary antibodies: NeuN (1:500; Millipore Inc., Billerica, MA, USA) for neuron detection and CD31 (1:300; R&D Systems) for vessel visualization. Neuronal and vessel density were quantified in 8 randomly selected microscopic fields of the spinal cord in areas adjacent to the epicenter of the compression injury.

### Preparation of spinal cord samples for SRμCT scanning 

To visualize the vasculature, rats were perfused transcardially with MICROFIL®, a low-viscosity radio-opaque polymer (Flow Tech, Inc., Carver, MA, USA), as previously described [29]. One cm of the thoracic spinal cord at level T10 was harvested presurgery and 1 and 6 months postsurgery (n=8 for each group) and fixed in 4% paraformaldehyde for 24βhours, and then maintained in 70% alcohol at room temperature until analysis. For neuronal network visualization, the rat was perfused transcardially with heparin and physiological solution, and the spinal cord tissue at the corresponding time point (n=8 for each group) was removed and cut to proper size consist the ventral horn or the dorsal horn of the spinal cord (0.5 mm × 0.5 mm × 0.5 mm) with a blade, followed by impregnation of with rapid Golgi and Golgi-CoA solutions (Sigma-Aldrich, USA) in alternating series, according to the manufacturer’s protocol. After SRμCT detection, the specimen was examined further by thick sectioning (20μm thickness) and examination by light microscopy.

### High-resolution SRμCT measurements

The scanning procedure was performed at the BL13W1 beamline of the Shanghai Synchrotron Radiation Facility (SSRF) equipped with a micro-CT apparatus (SRμCT). The spinal cord samples were put into a glass tube and fixed on the sample stage and examined using SRμCT. X-rays derived from an electron storage ring with an average beam current of 180 mA and an accelerated energy of 3.5 GeV were used for the measurements. The size of the beam was approximately 45 mm (horizontal) × 5 mm (vertical), and a double-crystal monochromator, with Si (111) and Si (311) crystals, was used to monochromatize the X-rays.

After X-rays penetrated through the sample, they were converted into visible light by a cleaved Lu_2_SiO_5_: Ce single-crystal scintillator (10 μm thickness). Projections were magnified by diffraction-limited microscope optics (×10 magnification for neuronal network visualization and ×2 magnification for vasculature visualization) and digitized with a high-resolution detector (ORCA Flash 4.0 Scientific CMOS, Hamamatsu K.K., Shizuoka Prefecture, Japan) with a physical pixel size of 0.74 μm for the neuronal network visualization and 3.25 μm for the vasculature detection). The samples were rotated continuously during the scanning, and 900 projection images were captured with an angular step size of 0.15° over 180° of rotation. The exposure time for each projection image was set to 150 ms. The distance between the detector and the sample was adjusted to 3 cm. Flat-field images with the X-ray illumination on the beam path without the samples and dark-field images with the X-ray illumination off were also collected during each acquisition procedure in order to correct the electronic noise and variations in the X-ray source brightness.

### 3D image reconstruction and quantitative analysis

The projected tomographic images were reconstructed using software developed by the SSRF to perform a direct filtered back-projection algorithm. Then, all the 2D slices of the spinal cord were processed by Amira software (version 6.01, FEI, USA) to obtain the reconstructed 3D images. Depending on the magnitude of X-ray absorption by the neurons and vasculature, differences in the gray values among tissues were determined, and microstructures of interest with a length of 1 cm for vascular analysis and a length of 0.5 mm for neuronal network analysis were extracted from the 3D models by segmentation. The 3D-rendered data were analyzed with the Image Pro Analyzer 3D (version 7.0, Media Cybernetics, Inc., Bethesda, MD, USA) to obtain quantitative data for the neuronal and vascular structures, as previously described[30, 31], including the vascular volume fraction, vessel number, vessel thickness, vessel bifurcation density, vessel segment density, vessel segment length of the spinal cord, central sulcal artery (CSA) angle, soma volume fraction, soma density, and neurite length.

### 3D neurovascular rendering

To optimize the visualization of the neurovascular network, high-resolution images obtained from SRμCT were rendered by coding each vessel branch, neurite and soma with a distinct color based on its connectivity, allowing the neurovascular distribution to be read more intuitively [32]. Additionally, the pseudocolor images were used to map the vascular surface to produce a 3D visual representation of the distribution of the spinal cord microvasculature correlated to the vessel thickness.

### Statistical analysis 

All quantitative data are presented as the mean?±?standard deviation. The 3D morphologic parameters of the vascular and neuronal network data were subjected to an arcsine transformation before statistical analysis to obtain a more normal distribution. One-way analysis of variance followed by the Dunn post hoc test was used to test the differences among groups at different time points postcompression. All analyses were carried out using SPSS version 19.0 (IBM Corp., Armonk, NY, USA), and P-values less than 0.05 were considered to indicate statistical significance.

**Figure 1.**
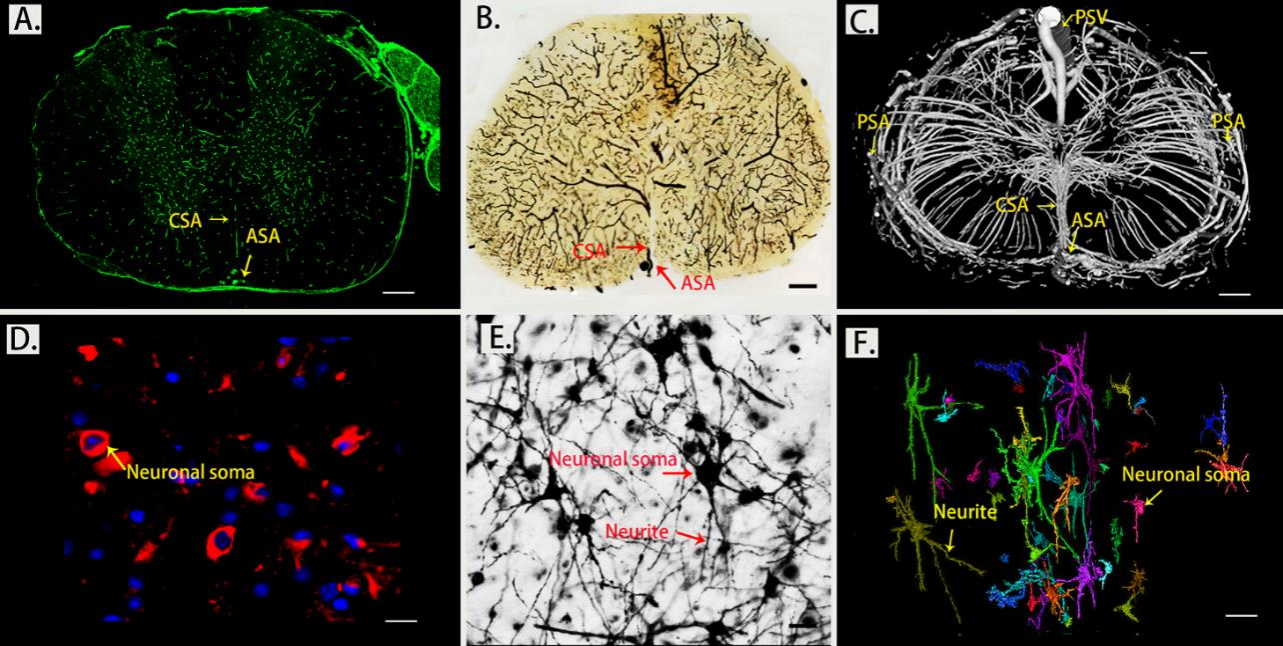
Morphology of the spinal cord microvasculature and neuronal network detected by a histological technique and SRμCT. (A) Immunofluorescence staining of transverse sections with CD31, scale bar = 250 μm; (B) Thick sections detected with light microscopy for vessel visualization, scale bar = 250 μm; (C) 3D SRμCT images of thoracic spinal cord microvasculature, scale bar = 250 μm; (D) Immunofluorescence staining of transverse sections with NeuN, scale bar = 20 μm; (E) Golgi stained neurons in transverse sections of the spinal cord examined by light microscopy, scale bar=20 μm; (F) Pseudocolored images of 3D structures of the intrinsic neuronal network of the spinal cord visualized with SRμCT, scale bar = 20 μm. (PSV?=?posterior spinal vein; PSA= posterior spinal artery; ASA?=?anterior spinal artery; CSA?=?central sulcal artery)

## RESULTS

### Visualization of the thoracic spinal cord vascular and neuronal network by using SRμCT and a histological method

The morphometric detection of neurons and vessels was simultaneously analyzed by both a histological method and SRμCT. A positive result for vessel visualization was defined as green staining located at the membrane of vascular endothelium. We used the well-known neuro marker NeuN to specifically detect neurons. NeuN reactivity (red staining) is found largely to be restricted to neuronal nuclei. The morphology of the spinal cord neurovascular network detected with immunofluorescence staining (Fig. 1A and D) was consistent with those of thick sections from SRμCT imaging with a light microscope, confirming the feasibility of using SRμCT for neurovascular detection. (Fig. 1B and E). SRμCT has great potential to detect 3D vascular and neuronal networks with pixel sizes down to micron-scale dimensions (Fig. 1C and F) and allows 3D analysis of the intricate neurovascular network in the spinal cord parenchyma.

**Figure 2.**
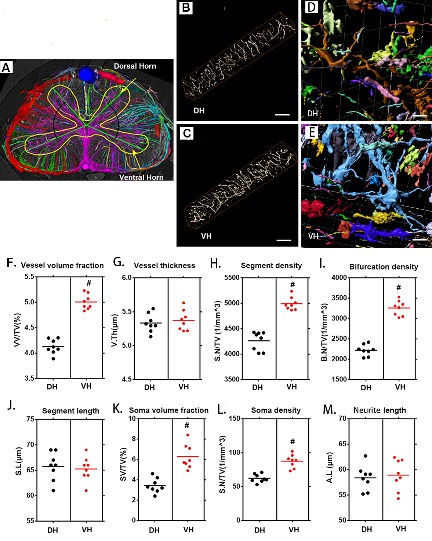
3D qualitative and quantitative characterization of the vascular and neuronal networks in the dorsal and ventral horn of the thoracic spinal cord. (A) 3D image of the spinal cord microvasculature obtained by SRμCT, with different color coding based on vessel connectivity. The yellow line outlines the gray matter. (B, D) 3D images of the vascular network and its corresponding neuronal network randomly selected from the dorsal horn. (C, E) 3D images of the vascular network and its corresponding neuronal network randomly selected from the ventral horn. (G-K) Quantification of the morphological characteristics of the vascular and neuronal networks in the ventral horn and dorsal horn of the thoracic spinal cord. In gray-matter structures of the spinal cord, vessel volume fraction, segment density, bifurcation density, soma volume fraction, and soma density were greater in the ventral horn than in the dorsal horn, and vessel thickness, segment length, and neurite length were similar. VH = ventral horn, DH=Dorsal horn. Scale bar = 20 μm. One-way analysis of variance followed by the Dunn post hoc test was performed. #*p*?<?0.05, significant difference among different groups. Scale bar = 20 μm.

**Figure 3.**
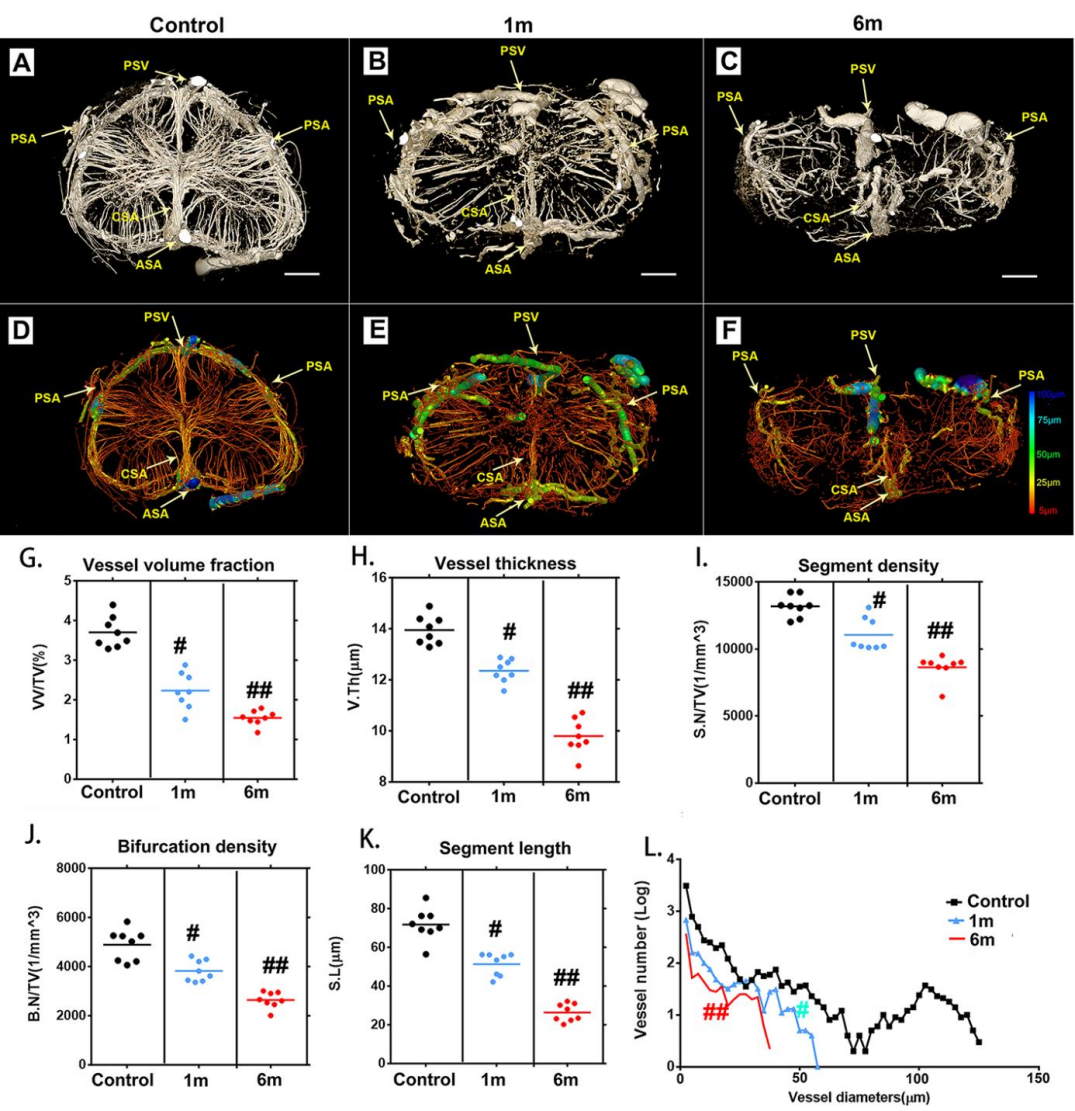
Characteristic 3D morphological alterations of the thoracic spinal cord microvasculature at different time points after surgery to induce chronic compression. (A-C) Representative 3D cross-sectional images of the spinal cord microvasculature after 1 and 6 months of chronic compression. (D-F) Pseudocolored image of the spinal cord microvasculature correlated with vessel thickness at the corresponding time point after compression surgery. (G-K) Quantification of morphological alterations in normal samples and after chronic thoracic spinal cord compression at different time points using network analysis. The vessel volume fraction (G), vessel thickness (H), segment density (I), bifurcation density (J) and segmental length (K) of the thoracic spinal cord significantly decreased after 1 month of chronic compression and worsened after 6 months, at the final follow-up. (L) Changes in the frequency distribution of vascular diameters in the normal and chronic compression groups after 1 and 6 months of compression. The pseudocolor bar in panel F indicates how vascular thickness was coded with different colors. (PSV?=?posterior spinal vein; PSA= posterior spinal artery; ASA?=?anterior spinal artery; CSA?=?central sulcal artery) Scale bar = 250 μm. One-way analysis of variance followed by the Dunn post hoc test was performed. ^#^*p*?<?0.05, significant difference between the control group and 1 month postcompression. ^##^*p*?<?0.01, significant difference between the control group and 6 months postcompression.

As presented in Figure 1C, one anterior spinal artery (ASA), two posterolateral spinal arteries (PSA), and one posterior spinal vein (PSV) were observed along the surface of the reconstructed 3D images. From the vertical view, the general outline of the spinal cord microvasculature could be separated into a CSA system and a peripheral arterial system. The large PSV was located on the dorsal surface of the spinal cord, while the ASA was located on the ventral surface of the spinal cord. We also found that the CSA and peripheral arteries were terminal branches and had no precapillary interconnections. The CSA originated from the ventral ASA and then entered the anterior median sulcus and branched into the bilateral gray matter at the anterior white commissure. Additionally, the gray matter where the penetrating branches of the CSA and that of the peripheral artery transversely and longitudinally anastomosed into a rich microvascular network was characterized by a unique butterfly shape (Fig. 1A and 1C).

Next, we turned our attention to the detection of the 3D distribution of neurite bundles and neuron soma using SRμCT. In Figure 1F, the neurons in spinal cord gray matter display unique topologies with various 3D shapes and sizes whose volumes could be quantified accurately. The color coding in the rendered image indicates the different sizes of neurons. Taking together, these data provide a valuable resource that can be used to further investigate the interaction of neurovascular networks in the central nervous system.

### Characterization of the 3D vascular and neuronal network of the thoracic spinal cord gray matter

Furthermore, we conducted a systematic qualitative and quantitative evaluation of the vascular and neuronal network in the dorsal and ventral horns of the thoracic spinal cord gray matter. The 3D angioarchitecture of the thoracic spinal cord obtained by SRμCT is vividly demonstrated in Fig. 2A. A unique butterfly shape was clearly visible and outlined the gray matter of the spinal cord in the 3D image, which included the dorsal horn and ventral horn. Moreover, the 3D angioarchitecture of the thoracic spinal cord was automatically labeled with different color codes using the previously mentioned algorithm [32], based on the vessel connections. Representative 3D images of the region of interest of the microvasculature and neuronal network in the gray matter of the dorsal horn and ventral horn were randomly selected. Local features, such as the 3D arrangement and relational features representing the connectivity of the vessel segments and color-labeled neuronal microanatomy that were derived from the dorsal and ventral horns, are separately displayed in Fig. 2B, 2C, 2D and 2E. Quantitative analysis revealed that vessel volume fraction, segments, and bifurcation density increased significantly in the ventral horn compared to the dorsal horn (Fig. 2F, H and I). However, the vessel thickness and segment length displayed no differences between the ventral and dorsal horns (Fig. 2G and J). Interestingly, we also found that the soma volume fraction and soma density were significantly higher in the ventral horn than in the dorsal horn (Fig. 2K and L). However, the neurite length in the neuronal network was similar in the ventral and dorsal horn (Fig. 2M).

### 3D visualization of microvasculature alterations after chronic compressive thoracic spinal cord injury

We next applied SRμCT to characterize 3D alterations of the microvasculature morphology of the thoracic spinal cord after chronic compression. The complete microvasculature changes in the thoracic spinal cord at T10 presurgery and 1 and 6 months after compression were respectively harvested and scanned by using SRμCT. The 3D digital maps of the cross-sectional views of microvasculature alterations during the chronic thoracic spinal cord compression process were vividly displayed via SRμCT (Fig. 3A-C). In the rendering stage, the vessel diameters were mapped with different color values (Fig. 3D-F). We obtained the full 3D structure of the spinal cord vascular network from arteries and veins down to the smallest capillaries.

One month after compression, intramedullary blood vessels with a diameter of approximately 50 μm disappeared at and around the lesion site (Fig. 3B, E and L). A progressive reduction of vessel numbers at various distances from the lesion site was observed during the ongoing compression phase at 6 months (Fig. 3C and F). The extramedullary blood vessels exhibited discontinuities at each time point after compression surgery (Fig. 3A-F). As this compression continued, the integrity and organization of blood supply in the lesion area were progressively destroyed. Several vascular parameters, including vessel volume fraction (Fig. 3G),

vessel thickness (Fig. 3H), vessel segment density (Fig. 3I), vessel bifurcation density (Fig. 3J), and vessel segment length (Fig. 3K) were decreased significantly after 1 month of compression. At 6 months of compression, the destruction of the vasculature in the spinal cord tissues became even worse (Fig. 3G-L). The vessels in the compressed spinal cord were significantly decreased compared to those in the normal spinal cord (Fig. 3L). All the morphology changes were revealed in detail through the spinal cord vascular network reconstruction in multiple perspectives.

**Figure 4.**
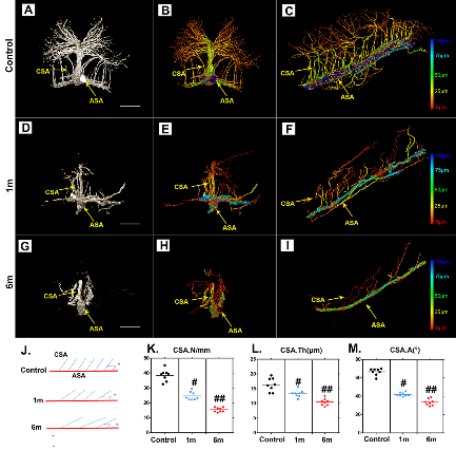
Characteristic 3D morphological alterations of the thoracic CSA after chronic compression. (A, D and G) Representative original 3D cross-sectional images of the CSA after 1 and 6 months of chronic compression. (B, C, E, F, H, and I) Pseudocolored images of the CSA correlated with vessel thickness at different time points after compression. (J) Schematic depiction of the CSA for quantification. (K, L, and M) Quantification of morphological alterations of the CSA in normal conditions and after 1 and 6 months of chronic thoracic spinal cord compression. The pseudocolor bar in panels C, F, and I indicate the vascular thickness coded with different colors. (ASA?=?anterior spinal artery; CSA?=?central sulcal artery). Scale bar = 250 μm. One-way analysis of variance followed by the Dunn post hoc test was performed. #*p*?<?0.05, significant difference between the control group and 1 month postcompression. ##*p*?<?0.01, significant difference between the control group and 6 months postcompression.

### 3D visualization of CSA alterations after chronic compressive thoracic spinal cord injury

The CSA branches into the parenchyma of the spinal cord at a regular angle and provides blood supply to the spinal cord. Several morphological parameters were employed to characterize pathological changes in the thoracic CSA after chronic compression. The original 3D images showed morphological alterations of the ASA and CSA at different compression times (Fig. 4A, 4D, and G). The blood vessels were then rendered with pseudocolors based on diameter (Fig. 4B, C, E, F, H, and I), which enhanced the visual quality of the vessels. To illustrate pathological changes of the angioarchitecture of the spinal cord, vascular quantification was conducted. A schematic diagram of the ASA and CSA showed morphological alterations at different time points after chronic thoracic spinal cord compression (Fig. 4J). The amount of CSA branches decreased significantly following chronic compression for 1 month compared to the normal spinal cord, with only a few CSA branches extending into the parenchyma at 6 months of compression (Fig. 4K). The diameter of the CSA also showed a decrease after 1 month of compression and became worse after 6 months of compression (Fig. 4L). Interestingly, the normal average intersection angle between the CSA and ASA is 64°?±3.9°. However, the CSA became distorted and fell back toward the caudal orientation at 6 months of compression, with an average angle of 42°?±?5.6°, showing a significant decrease (Fig. 4M).

**Figure 5.**
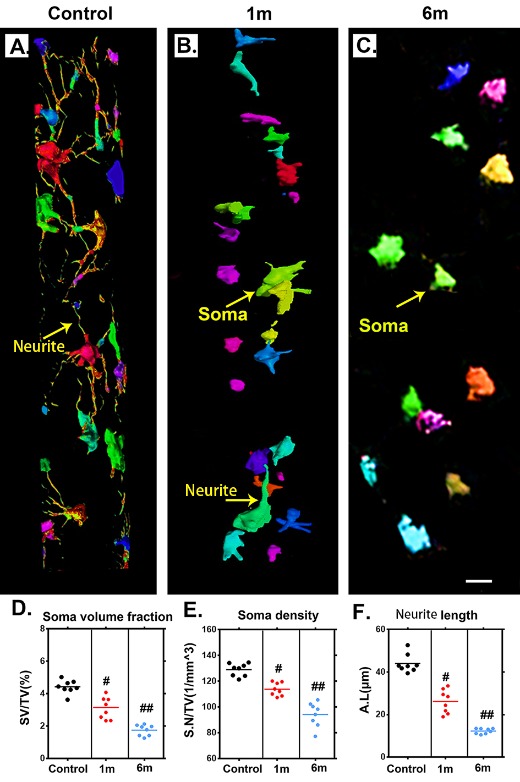
Characteristic 3D morphological alterations of the neuronal network in the thoracic spinal cord after chronic compression. (A-C) Randomly selected 3D images of the neuronal network in the ventral horn before chronic compression and after 1 and 6 months of chronic compression. (D-F) Quantification of morphological alterations of the neuronal network in normal samples and after 1 and 6 months of chronic thoracic spinal cord compression using network analysis. The soma volume fraction, soma density, and neurite length decreased significantly and were even worse at the final follow-up. Scale bar = 20 μm. One-way analysis of variance followed by the Dunn post hoc test was performed. #*p*?<?0.05, significant difference between the control group and 1 month postcompression. ##*p*?<?0.01, significant difference between the control group and 6 months postcompression.

**Figure 6.**
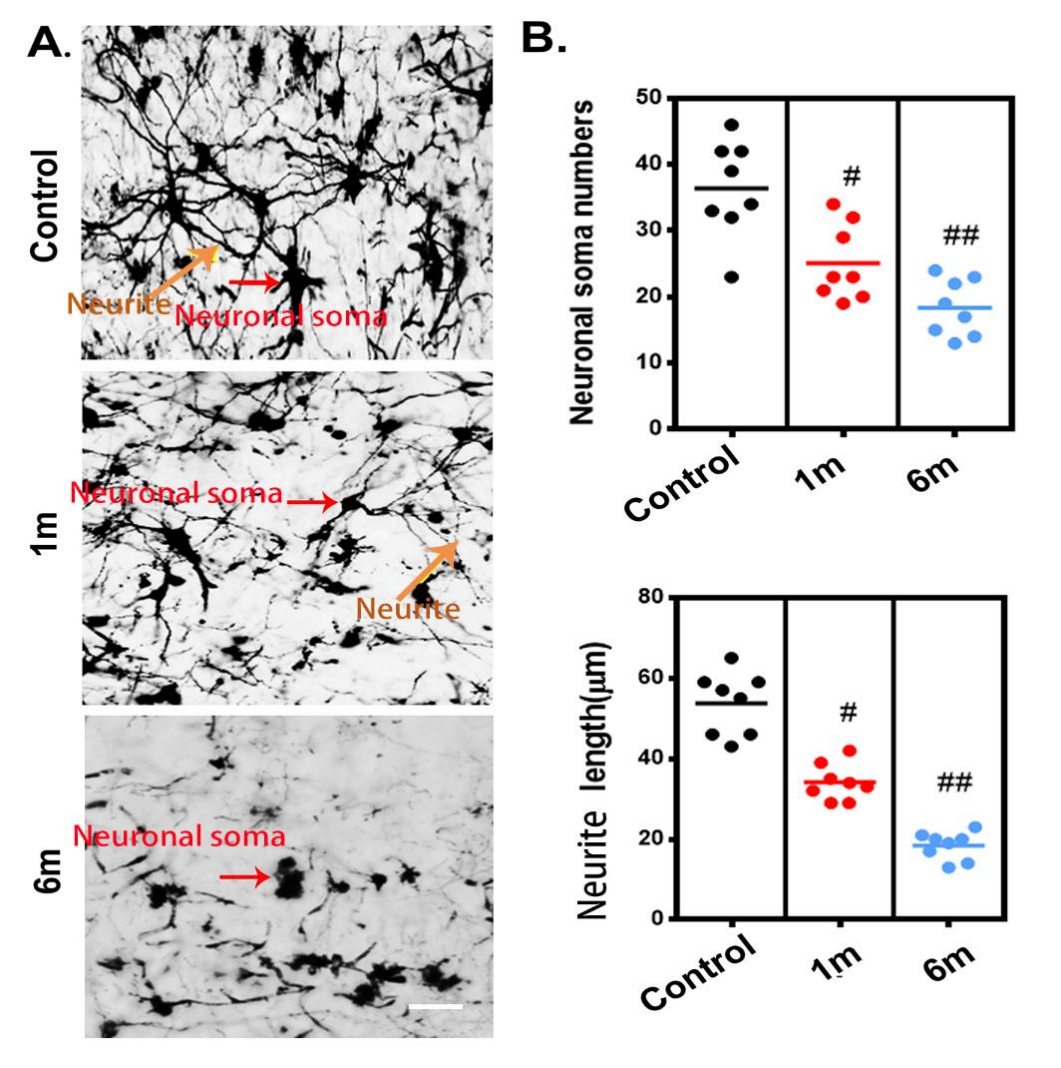
The histological morphological alterations of the neural network in the thoracic spinal cord after chronic compression, as detected using Golgi staining. (A) Representative images of the neural network randomly selected from longitudinal sections (30 μm) of the spinal cord in the control group and after 1 and 6 months of chronic thoracic spinal cord compression. Scale bar= 50 μm. (B) Quantification of morphological alterations of the neuronal network in normal samples and after 1 and 6 months of chronic thoracic spinal cord compression. A one-way analysis of variance followed by the Dunn post hoc test was performed. #*p*?<?0.05, significant difference between the control group and 1-month postcompression. ##*p*?<?0.01, significant difference between the control group and 6 months postcompression.

### 3D visualization of neuronal network alterations after chronic compressive thoracic spinal cord injury

To trace morphological changes in the neuronal network after chronic thoracic spinal cord compression, toward a comprehensive understanding of the pathological mechanism, one region of interest in the ventral horn of the spinal cord was selected at random for further analysis. We extracted 3D structural data of the neuronal network at different time points after chronic thoracic spinal cord compression. The morphology and spatial locations of neurons and the traces of neurites in the spinal cord could be vividly visualized in both normal and compressed spinal cord (Fig. 5A-C). The colors indicate different sizes of neuronal soma. The architecture of the neurons and neurites has been destroyed by one month of compression (Fig. 5B), followed by a dramatic decrease at 6 months after compression (Fig. 5C). A quantitative analysis demonstrated significant decreases of the soma volume fraction, soma numbers, and neurite length at 1 month of compression and even more notably at 6 months of compression (Fig. 5D-F). The change in the architecture of the spinal cord neuronal network visualized with SRμCT was similar to the pattern observed in compressed spinal cord via Golgi staining (Fig. 6A and B).

### Visualization of neurovascular network alterations after chronic compressive thoracic spinal cord injury with a histological method

**Figure 7.**
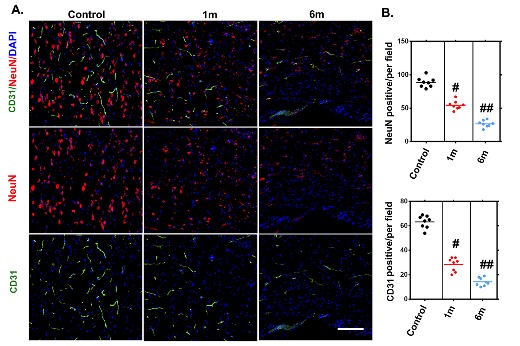
Histologically visualized morphological alterations of vascular and neuronal networks of the thoracic spinal cord after chronic compression. (A) Representative immunofluorescence images of the vascular and neuronal network, randomly selected from longitudinal sections of the spinal cord before and after 1 and 6 months of chronic compression. (B) Quantification of vascular and neuronal network changes in normal samples and after 1 and 6 months of chronic thoracic spinal cord compression. The NeuN- and CD31-positive cell numbers significantly decreased and were even worse at the final follow-up. Scale bar = 20 μm. One-way analysis of variance followed by the Dunn post hoc test was performed. #*p*?<?0.05, significant difference between the control group and 1 month postcompression. ##*p*?<?0.01, significant difference between the control group and 6 months postcompression.

We applied immunofluorescence staining to visualize the neurovascular network alterations after chronic compressive thoracic spinal cord injury. We observed gradual reduction of vessel density in the compressed thoracic spinal cords sections at different time points measured by immunofluorescence staining, confirming the feasibility of using SRμCT for vessel detection in pathologic conditions (Fig. 7A and B). The morphology changes in the spinal cord neuronal network also showed the same pattern in the compressed spinal cord through immuneo-fluorescence staining as those detected by SRμCT, indicating that SRμCT has the sensitivity to detect neuronal architecture changes in the spinal cord (Fig. 7A and B). Furthermore, the trend of changes in microvasculature after compression was consistent with the results observed with neural staining.

## DISCUSSION

The neurovascular unit (NVU) has a specialized 3D microstructure that is an interdependent system composed of neuronal and vascular networks, along with supporting cells [33]. 3D investigations of ultrastructural changes of the NVU during pathological processes may contribute to the development of novel therapeutic targets. In the present study, SRμCT imaging allowed 3D visualization of neuronal and vascular changes during the thoracic spinal cord compression process within unsanctioned organs. High-resolution 3D imaging combined with network analysis can substantially improve our understanding of pathological changes in neurovascular architecture after injury, which is beneficial for developing novel therapies to improve neurological functional recovery.

The vasculature is the core anatomical structure of the NVU. Many studies have demonstrated that neurovascular diseases are associated with vascular alterations [33]. In the current study, in which we present a detailed 3D digital anatomic map of the angioarchitecture of the healthy thoracic spinal cord, we found that the spinal cord vessel branches were numerous and deeply enclosed in the neural parenchyma. The CSA, which is derived from the ASA, supplies two-thirds of the spinal cord [34]. In 3D views, the features of the CSA in the thoracic spinal cord were vividly displayed. Our results showed that the CSA at the thoracic region emerged with a smaller diameter before penetrating the parenchyma. Due to the poor blood supply in the thoracic region, the compromised of local blood flow creates a higher risk of ischemia, which is harmful to the thoracic spinal cord [35-37]. Previous studies have shown an approximately 4% incidence of spinal cord infarction following the repair of aneurysms in the descending thoracic aorta [38-40]. The findings of this study also indicate that chronic thoracic spinal cord compression will lead to disastrous neurological impairment without prompt surgical decompression. Our results provide valuable biological imaging evidence to elucidate the underlying mechanism of the vascular hemodynamics of the thoracic spinal cord.

In the central nervous system, neurons are extremely sensitive cells and engage in crosstalk with endothelial cells [41]. In the present study, we demonstrate the 3D morphology of neuronal systems of ex vivo rat spinal cord. Within the gray matter, the density of the neurons exhibited specific patterns. We observed denser distributions of neuron soma in the ventral horn of the gray matter than in the dorsal horn. In this region, the vasculature of the spinal cord is served by abundant capillary beds that supply oxygen and nutrients to meet the substantial nutrient demands for neuronal survival [38]. This arrangement reflects the fact that the motoneuron bodies in the ventral horn have greater metabolic requirements than the sensory neurons in the dorsal horn of the spinal cord.

The concept of the NVU highlights the importance of interactions between the vasculature and the neuronal network [42]. Therefore, simultaneously imaging the 3D ultrastructural features of the NVU after chronic thoracic compression is essential to clarify the interactions between the neurons and vasculature and to determine which components undergo changes that are responsible for thoracic neural deficits. Furthermore, the visualization of NVU changes will facilitate establishment of precise regenerative strategies for chronic thoracic spinal cord injuries. In this study, changes in the neuronal and vascular networks during the thoracic spinal cord compression process were demonstrated for the first time. We observed structural damage of the NVU in the thoracic spinal cord following compression. Interruption of blood supply of the spinal cord led to irreversible neuronal loss in the ischemic region. This observation is consistent with previous transmission electron microscopy studies showing ultrastructural changes of NVUs after chronic cervical cord compression [19]. However, no previous data focused on 3D ultrastructural changes of NVUs after thoracic spinal cord compression. The specialized network of vasculature delivers oxygen and nutrients throughout the nervous system, supporting neuronal survival and homeostasis [38]. Direct physical compression of the spinal cord caused the CSA to bend within the lesion epicenter, initiating intramedullary ischemia rostrally and caudally. More importantly, neurons in the ventral horn were influenced by changes in their microenvironment and displayed irregular and disorganized dendrites. The abnormality of the CSA after compression leads to spinal cord ischemia, which could be the predominant factor contributing to the onset and progression of neurological dysfunction.

Our data indicated that the preservation of spinal cord vasculature function is an essential component of ischemic tolerance after physical compression. The microvasculature and neurons respond simultaneously to ischemic insults. Combining neuroprotection and vasoprotection may be a valuable strategy for treating chronic spinal cord compression injuries.

A prerequisite for studying the biological response of vascular and neuronal remodeling is the development of methods that enable visualization and quantification of the 3D structural properties of neurovascular networks. A recently developed approach for this purpose is hierarchical network analysis in combination with high-resolution 3D SRμCT imaging [43, 44]. SRμCT allows precise visualization of the 3D morphology of the ASA and CSA in selected anatomical regions of interest, and this information is captured nondestructively at a high resolution with sectioning. 3D visualization of the entire spinal cord NVU simultaneously demonstrated the location and trajectory of the vascular and neuronal networks, and such information can be used to provide new insights for studies of interactions between neurons and the vasculature. However, characterising tissue structure by conventional histological sectioning frequently induces tears, compressions and folds, which may lead to data misinterpretation in studies of vessel and neurite regeneration. In our study, 3D vessel network analysis was employed to characterize the morphometry of vascular and neuronal networks of the rat spinal cord. New structural indices calculated using network analysis from the digital 3D datasets obtained by SRμCT provide a deeper understanding of the complex neurovascular network repair and reorganization process. It is important to note that we used Golgi staining combined with SRμCT to visualize the neuronal networks in the gray matter of the spinal cord. However, it is difficult to differentiate neurons by cell type. The 3D morphology of the microvascular network and neuronal soma in the normal spinal cord has been visualized previously [45]. Nonetheless, simultaneous submicrometric 3D imaging of neuronal and vascular alterations after chronic thoracic spinal cord compression remains a challenging task. To explore the relationships and interaction between the vascular and neuronal systems during chronic thoracic spinal cord compression, we plan to apply SRμCT to simultaneous 3D imaging of neuronal and vasculature alterations after longer periods of chronic compression.

### Conclusion

In summary, we present a high-resolution method based on SRμCT to investigate 3D microstructural changes of vascular and neuronal networks after chronic thoracic spinal cord compression. Our imaging data reveal further insights into the pathological changes of the NVU in response to chronic compression of the spinal cord. This work offers a potential novel platform to explore the relationship between the vasculature and neurons, promoting the development of a regenerative strategy for the treatment of neurovascular diseases.
